# Health system barriers and pathways to strengthening early detection of pediatric type 1 diabetes in low- and middle-income countries: a systematic review

**DOI:** 10.1186/s12889-026-28810-2

**Published:** 2026-08-03

**Authors:** Mehrdad Yadegari, Atousa Bonyani, Ashley Mitchell, Alden Hooper Blair, Ric Bonnell, Tracy Kuo Lin, Christopher Carpenter

**Affiliations:** 1 Department of Social and Behavioral Sciences, Institute for Health and Aging, School of Nursing, University of California, San Francisco, 490 Illinois St., San Francisco, CA 94158 USA; 2 Institute for Global Health Sciences, University of California, San Francisco, CA USA; 3 Global Action in Nursing (GAIN), Institute for Global Health Sciences, University of California, San Francisco, CA USA; 4 Burnett School of Medicine at Texas Christian University, Fort Worth, TX USA; 5 Department of Pediatrics, School of Medicine, University of California, San Francisco, CA USA

**Keywords:** Type 1 diabetes mellitus, Early diagnosis, Low- and middle-income countries, Primary health care

## Abstract

**Background:**

Type 1 diabetes mellitus (T1DM) affects approximately 1.2 million children and adolescents globally. Delayed diagnosis in low- and middle-income countries (LMICs) is a key factor for high morbidity and mortality. The available evidence suggests systemic barriers across the diagnostic pathway. This systematic review aims to synthesize evidence on facilitators and barriers to early detection of pediatric T1DM in LMICs.

**Methods:**

We conducted a systematic review following Preferred Reporting Items for Systematic Reviews and Meta-Analyses (PRISMA) guidelines. PubMed, Web of Science, Embase and Cochrane Library were searched in February and March 2026. Additional targeted grey literature searches of the World Health Organization IRIS repository, and the International Diabetes Federation website were conducted separately. Studies were eligible if they examined interventions, implementation strategies, or health system approaches to improve timely detection and diagnosis of pediatric T1DM in LMICs. Two reviewers independently screened studies in Covidence. Risk of bias was assessed, and findings were synthesized using descriptive and thematic analysis according to the WHO health system building blocks framework.

**Results:**

Nine studies met the inclusion criteria. Barriers were most frequently identified in service delivery, the health workforce, and access to diagnostics. Early detection was largely passive and concentrated in higher-level facilities, with limited integration into primary care. Fragmented referral pathways, weak linkage to care, and gaps in provider knowledge contributed to delayed diagnosis. Several approaches, including integration with existing programs and community-based strategies, demonstrated promise; however, they were not implemented at scale and remained fragmented.

**Conclusion:**

Early detection of pediatric T1DM in LMICs remains challenging and is primarily constrained by health system limitations rather than the absence of diagnostic tools. Evidence evaluating implemented strategies remains limited. Addressing these delays requires coordinated, system-level strategies that strengthen primary care capacity, expand access to basic blood glucose testing at first-contact points, and improve referral and follow-up across the care pathway. The limited number of studies evaluating implemented interventions highlights a critical need for research on scalable, integrated approaches in resource-constrained settings.

**Trial registration:**

CRD420261330542 (March 03, 2026).

**Supplementary Information:**

The online version contains supplementary material available at https://doi.org/10.1186/s12889-026-28810-2.

## Introduction

Type 1 diabetes mellitus (T1DM) is a chronic autoimmune condition affecting approximately 1.2 million children and adolescents worldwide [1]. T1DM requires lifelong insulin therapy for survival and careful ongoing management to prevent complications [2]. Despite advances in prevention strategies and efforts to promote earlier diagnosis, the incidence of T1DM continues to rise globally [3]. Without timely diagnosis and treatment, there is a high risk of acute complications such as diabetic ketoacidosis (DKA), a life-threatening metabolic emergency, and a major contributor to morbidity and mortality [4]. These challenges are exacerbated in low- and middle-income countries (LMICs), where children are more likely to present late and with more severe disease, with reported rates of DKA at diagnosis often exceeding 50% [5, 6]. Late presentation not only increases the risk of death, but also results in greater healthcare utilization, including intensive care admissions and prolonged hospital stays. These consequences place additional strain on already resource-limited systems [7–10]. Importantly, many of these adverse outcomes are preventable with timely diagnosis, positioning early detection as a high-impact, system-level opportunity for reducing avoidable morbidity, mortality, and health system costs in resource-constrained settings.

The diagnosis of T1DM is based on standard biochemical criteria for hyperglycemia. In children, diagnosis is most commonly established using blood glucose measurement to test for T1DM and can be confirmed with an elevated random blood glucose measurement in the presence of symptoms such as weight loss, polyuria, or polydipsia [11, 12]. Additional biomarkers, such as autoantibodies or C-peptide, may support the classification of diabetes type, but are not required for initial diagnosis [13]. In clinical practice, hyperglycemia is typically measured using point-of-care glucometers with test strips, laboratory-based plasma glucose assays, or urine dipsticks checking for glucose and ketones [14–16]. Most of these diagnostic tools are low-cost, [17, 18] require minimal infrastructure, and do not depend on complex laboratory systems or cold-chain storage, which makes them feasible for use at the primary care level and other first-contact points within the health system [19–21]. Despite the availability of simple, low-cost diagnostic tools, early detection of T1DM remains inconsistent, suggesting that gaps are driven less by technological constraints and more by health system and implementation challenges.

Timely diagnosis of T1DM among children remains suboptimal in many LMIC settings [22]. From a health systems perspective, challenges are layered across key domains, including service delivery, health workforce, health information systems, access to essential diagnostics and technologies, financing, and governance [23]. Breakdowns at any stage of the diagnostic pathway—from initial symptom recognition to confirmation of diagnosis and linkage to care—can result in missed opportunities for timely detection and intervention [24, 25].

Existing evidence on early detection is fragmented across these domains and across stages of the care pathway. Previous studies have examined awareness campaigns and models of pediatric diabetes care with evidence mostly derived from high-income settings [26–28]. There has been limited attention to LMIC-specific interventions, which impedes the ability of decision-makers to identify feasible and context-appropriate strategies for T1DM screening and detection in these higher-risk settings [29, 30]. This fragmented evidence base leaves policymakers and funders making decisions on where to invest—whether in workforce training, diagnostic access, service delivery models, or community-based approaches—often without clear synthesized guidance on which strategies are most feasible, scalable, cost-effective, and impactful in LMIC settings. As a result, investment decisions are often made with limited information on how interventions interact across diagnostic pathways or how they can be embedded within the existing health system. Addressing early detection of T1DM, therefore, requires a systems-oriented approach that identifies not only barriers but also coordination strategies.

We carried out a review of factors influencing the timely detection and diagnosis of T1DM among children and adolescents in LMICs. We synthesized evidence across the early detection pathway and leveraged the World Health Organization (WHO) health systems building blocks framework to identify key system-level facilitators and barriers. Our aim was to generate a more integrated, system-oriented understanding of early detection that can inform policy, guide resource allocation, and support the design of feasible and scalable future implementation strategies in resource-constrained settings.

## Methods

This systematic review was conducted following the Preferred Reporting Items for Systematic Reviews and Meta-Analyses (PRISMA) guidelines [31]. The protocol was registered in the International Prospective Register of Systematic Reviews (PROSPERO; registration number CRD420261330542; registered on March 03, 2026). The search strategy was finalized before protocol registration. Database searches were initiated in February 2026, and the protocol was registered on 3 March 2026 while title and abstract screening was underway, before completion of study selection, data extraction, quality assessment, and data synthesis.

No substantive changes were made to the search strategy following registration. However, during screening, the eligibility criteria were broadened to include additional study designs and broader pediatric populations because of the limited evidence base. This methodological deviation is described below. We searched PubMed (MEDLINE), Web of Science Core Collection, and Embase between February and March 2026. Additional searches of the Cochrane Library and targeted grey literature sources, including the WHO IRIS repository and the International Diabetes Federation (IDF) website, were subsequently conducted. The targeted grey literature searches were conducted separately from the bibliographic database searches. The complete search strategies for all sources are provided in Supplement 1. Ethical approval was not required for this desk-based review.

### Eligibility criteria

We aimed to identify and synthesize evidence on interventions designed to improve the timely detection and diagnosis of pediatric T1DM in LMICs. As such, the search strategy combined four key concepts: (1) type 1 diabetes and related conditions, (2) early detection and diagnosis, (3) interventions and implementation, and (4) low- and middle-income country settings. Controlled vocabulary (e.g., MeSH terms) and free-text terms were used. No date restrictions were applied toward capturing all available evidence. We defined early detection as processes occurring from pre-symptom recognition to confirmed diagnosis, including routine screening, caregiver recognition, initial care-seeking, diagnostic evaluation, and confirmation. The search strategy was intentionally broad to capture both interventional studies and health systems research related to early diagnosis. Studies were eligible for inclusion if they examined interventions, implementation strategies, or health system approaches to improve screening, early detection, or diagnosis of T1DM among children and adolescents (0–18 years) in LMICs [32]. We defined LMIC according to the World Bank classification (2024) [33]. Due to the limited number of studies specifically focused on early detection of pediatric T1DM in LMICs, the eligibility criteria were broadened during the screening process to capture relevant system-level evidence. Specifically, we included studies with broader age groups where findings were applicable to children and adolescents, studies addressing pediatric diabetes without explicit differentiation of diabetes type, and descriptive or case-based studies that provided relevant insights into diagnostic processes. This modification was made after protocol registration in response to the limited available evidence and is acknowledged as a methodological limitation.

We excluded studies that 1) focused solely on post-diagnosis management (such as glycemic control, insulin delivery, or long-term complications, unless they were directly linked to timing of diagnosis) 2) focused exclusively on adult populations, 3) focused on type 2 diabetes or other conditions, 4) were non-primary research (including editorials, opinion pieces, conference abstracts, protocols, and guidelines), and 5) were published in non-English languages. Given the limited availability of primary studies in this area, we included one narrative study that provided relevant insights into diagnostic processes in resource-constrained settings.

### Study selection

All identified titles and abstract records were imported into Covidence systematic review software [34] and duplicate records were removed. Titles and abstracts and the full texts of potentially eligible studies were screened by two independent reviewers. Discrepancies at both stages were resolved through discussion until consensus was reached. Inter-rater agreement between reviewer pairs was assessed at both screening stages using Cohen's kappa and percent agreement. During full-text review, the reference lists of included studies and relevant reviews were also screened to identify additional studies using a snowballing approach.

### Quality assessment

We used the Mixed Methods Appraisal Tool (MMAT) to assess the risk of bias of included studies [35]. For narrative reviews, we used the Scale for the Assessment of Narrative Review Articles (SANRA) to assess quality [36]. Both tools were applied according to their respective guidelines to ensure appropriate appraisal across differing study designs.

### Data extraction

Data were extracted into Microsoft Excel using a 29-item matrix predetermined by the study team. We extracted information on study characteristics (e.g.,country, setting, study design, and sample size), population, intervention details, and outcomes. In addition, we extracted information on implementation characteristics, including reported barriers and facilitators, as well as measures of feasibility, acceptability, and uptake where available. Finally, studies were categorized by intervention type and mapped to stages of the early detection pathway, including awareness, first contact, screening, diagnosis, or linkage to care.

### Data synthesis and analytical framework

Findings were analyzed using the WHO health system building blocks: service delivery, health workforce, health information systems, access to essential medicines and technologies, financing, and leadership and governance [37]. Each study’s reported interventions, barriers, and outcomes were categorized according to the relevant health system building blocks to identify system-level strengths and gaps. Interventions and barriers were summarized along the early detection pathway, including awareness and symptom recognition, initial contact with the health system, access to diagnostic testing, confirmation of diagnosis, and linkage to care. Given the heterogeneity in study designs, interventions, and reported outcomes, a narrative synthesis approach was used [37, 38]. Studies were grouped by intervention type and summarized descriptively. Thematic analysis was then conducted using an inductive approach, in which key concepts related to barriers, facilitators, and system-level gaps were identified, coded, and grouped into themes affecting early detection, independent of the analytical framework. The synthesized themes were subsequently mapped to both the WHO health system building blocks and stages of the diagnostic pathway; this mapping is presented in Table S1 (Supplementary File 2). Quantitative outcomes, where reported, were summarized using descriptive statistics, including proportions and frequencies, and measures of central tendency where available. This analytical approach was designed to generate decision-relevant insights on how early detection strategies can be integrated within existing health systems. To evaluate the influence of lower-level evidence on the review findings, we conducted a sensitivity analysis by repeating the narrative synthesis after excluding the two preprint studies and the single-patient clinical narrative report. We compared the resulting themes with the primary synthesis to determine whether the overall interpretation changed.

## Results

**Fig. 1:**
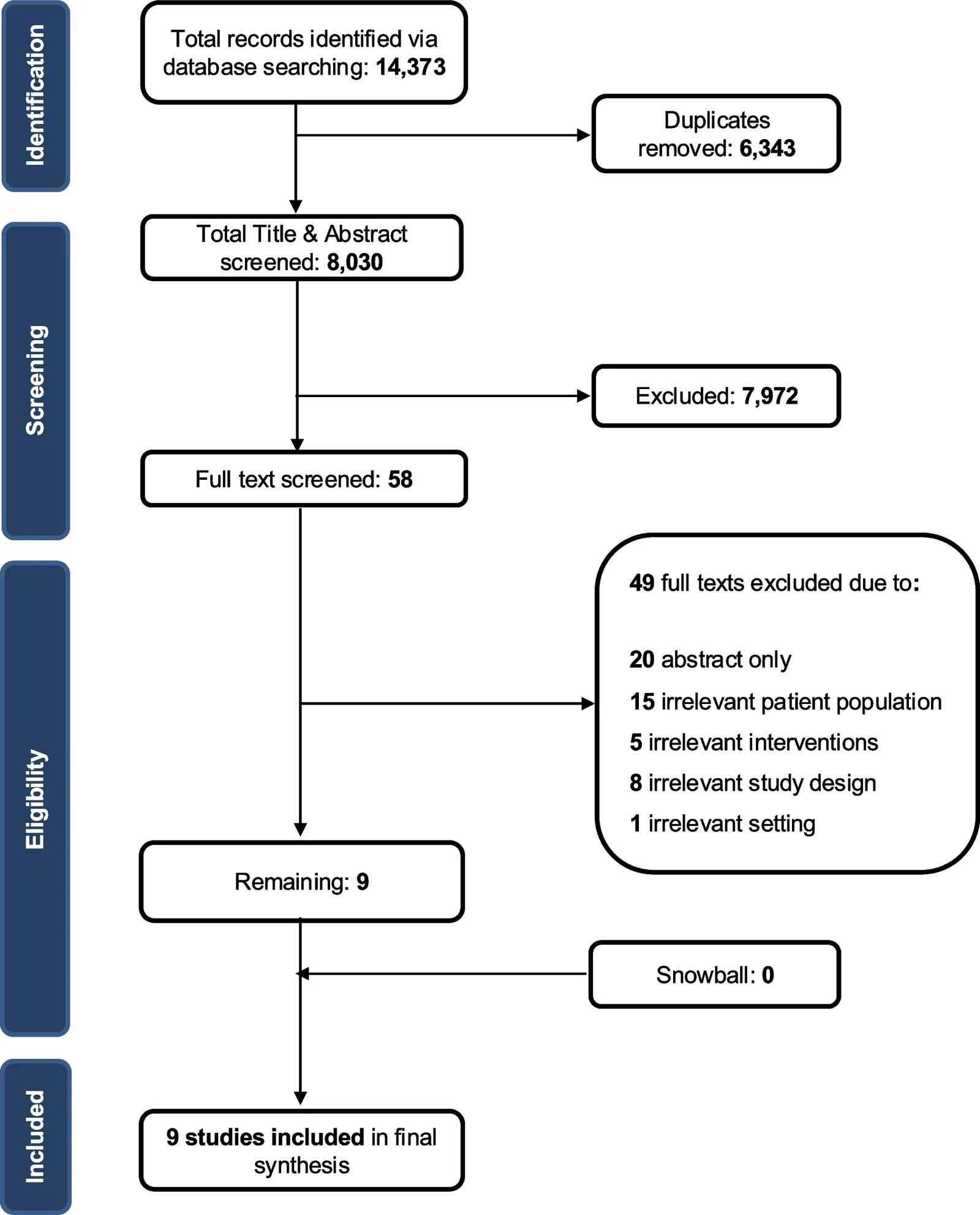
PRISMA flow diagram outlining the study selection process. Targeted grey literature searches did not identify any additional eligible primary studies and are therefore not represented in the PRISMA flow diagram

A total of 14,373 records were identified through structured searches in PubMed (*n =* 3,888), Web of Science (*n =* 3,335), Embase (*n =* 6,517), and Cochrane Library (*n =* 633). After removing 6,343 duplicates, 8,030 records were screened by title and abstract, of which 7,972 were excluded. A total of 58 full-text articles were assessed for eligibility. Of these, 49 studies were excluded due to abstract-only availability (*n =* 20), irrelevant patient population (*n =* 15), irrelevant intervention (*n =* 5), irrelevant study design (*n =* 8), or an irrelevant setting (*n =* 1). No additional relevant studies were identified during the screening of reference lists. Inter-rater agreement between reviewer pairs at title/abstract screening was fair to substantial (Reviewer 1–2: κ = 0.24, 98% agreement; Reviewer 1–3: κ = 0.50, 99.5% agreement; Reviewer 2–3: κ = 0.12, 98.6% agreement). At full-text screening, agreement was moderate for one reviewer pair (Reviewer 2–3: κ = 0.43, 72% agreement) and could not be meaningfully assessed for a second pair due to a limited number of shared decisions (Reviewer 1–2: *n =* 4). Ultimately, nine studies were included in the final synthesis (Fig. 1).

### Quality assessment

The methodological quality of the included empirical studies was assessed using the MMAT, while the narrative review was evaluated using the SANRA. Across the eight empirical studies, MMAT scores ranged from 5 to 7 out of 7, indicating moderate to high methodological quality. All studies clearly stated their research questions and collected data appropriate to address them. The mixed-methods studies demonstrated adequate integration of qualitative and quantitative components but showed limitations in addressing divergences between findings and in meeting quality criteria for both methodological traditions. Among the quantitative non-randomized studies, most demonstrated strong methodological rigor, including appropriate measurements, complete outcome data, and adequate consideration of confounding.

The single narrative review was assessed separately using SANRA and scored 10 out of 12, indicating generally high methodological quality. Its primary limitation was the absence of a clearly described literature search strategy. No studies were excluded based on quality concerns. Details of the quality and risk of bias assessments are presented in Tables S2 and S3 in Supplement 3.

### Study characteristics

The nine included studies were conducted across LMIC countries in sub-Saharan Africa, South Asia, Latin America, and the Middle East [39–47]. Study designs were heterogeneous and included cross-sectional studies (*n =* 4), [39, 41, 45, 46] retrospective analyses of routine or programmatic data (*n =* 2), [42, 43] mixed-methods studies (*n =* 2), [40, 44] and one clinical narrative review (*n =* 1) [47]. Sample sizes varied widely, ranging from a single-patient clinical narrative report [47] to national-level data involving over 256,000 individuals [42]. The studies examined health system readiness, provider knowledge, care cascade performance, implemented and pilot approaches to improve detection, and proposed diagnostic approaches [39–47]. Service delivery (*n =* 9) and health workforce (*n =* 9) were the most frequently addressed components, followed by access to diagnostics and technologies (*n =* 8) and health information systems (*n =* 6), while leadership and governance (*n =* 6) and financing (*n =* 4) were less frequently examined (Table 1).

**Table 1:** WHO health system building blocks influencing timely detection of pediatric T1DM in LMICs

WHO Health System Building Block	Facilitators	Barriers	Resources/Enablers	Implementation Insights & Implications
Service Delivery	- Integration with existing programs (e.g., TB-DM screening within TB treatment pathways) - Community-based approaches (home-based self-testing, mobile screening camps) - Partial decentralization of services	- Centralized care at tertiary facilities - Weak referral pathways and follow-up systems - Fragmented care continuum - Limited primary care capacity - Delays between presentation and diagnosis - Loss to follow-up across care pathways	- Existing vertical programs (TB, HIV) - Community outreach platforms - Primary care infrastructure (underutilized)	Early detection is largely passive and facility-based. Leveraging existing programs and decentralizing services can improve access, but weak referral and follow-up systems limit continuity of care
Health Workforce	- Clinical exposure during training - Targeted training approaches - Specialist support (where available)	- Knowledge gaps in early symptom recognition - Limited clinical exposure - Variability in training - Shortage of trained providers	- Medical education systems - Training programs - Specialist networks (limited)	Early detection depends heavily on frontline providers. Strengthening training and task-sharing is critical to improving early recognition
Health Information Systems	- National laboratory systems (e.g., NHLS) - Digital data platforms and mobile tools - Real-time reporting systems in some programs	- Fragmented or paper-based systems - Poor data integration across levels of care - Limited patient tracking and follow-up	- Digital tools (apps, MIS systems) - National databases (where available)	Data systems exist but are often not linked to clinical decision-making or referral pathways. Integration of data with care pathways is essential for improving detection and follow-up
Access to Essential Medicines & Technologies	- Availability of basic diagnostics in some settings - Emerging technologies (POCT, self-testing, AI models) - Technology-enabled screening approaches	- Limited availability of glucose testing and HbA1c - Supply chain disruptions - High cost of technologies - Limited access at primary care level	- Point-of-care devices - Laboratory infrastructure - Digital and AI tools (pilot stage)	The gap lies not only in availability but in routine use. Expanding access to low-cost diagnostics at first-contact points is essential to reduce reliance on clinical judgment and improve early detection
Financing	- Inclusion of diabetes services in UHC packages (in some settings) - Insurance schemes covering diagnostics and treatment - Donor and NGO support programs	- High out-of-pocket costs - Underfunded NCD programs - Reliance on external funding - Limited coverage for diagnostics and technologies - Transport costs and access constraints - Caregiver-related barriers to accessing services	- UHC frameworks - Insurance systems - External funding mechanisms	Financial and logistical barriers limit access to diagnosis and follow-up. Sustainable financing and reduced access costs are critical for improving early detection
Leadership & Governance	- National NCD strategies and policy frameworks - Integration opportunities within existing programs - Stakeholder engagement and advocacy	- Weak implementation of policies - Lack of dedicated early detection strategies - Fragmented program coordination - Dependence on vertical programs	- National health policies - Existing program infrastructure (TB, HIV) - Partnerships with NGOs	Policy presence does not translate into implementation. Stronger coordination and integration of early detection into primary care systems are needed

### Preprint studies

Two of the nine included studies were available only as preprints at the time of this review [42, 45]. These comprised a national T1DM care cascade analysis using South Africa’s National Health Laboratory Service database [42] and a community-based home self-testing study conducted in Kenya, South Africa, and Zambia [45]. Given the limited evidence base, these studies were retained in the primary synthesis but were evaluated separately in the sensitivity analysis.

### Service delivery

Service delivery models played a central role in shaping early detection pathways. Across studies, diabetes detection was predominantly facility-based and often centralized at secondary or tertiary care levels. In many settings, diagnosis occurred only after patients presented with advanced symptoms. Some studies described more proactive or decentralized approaches embedded within existing service delivery platforms. For example, a bi-directional TB–DM screening program demonstrated that integrating diabetes screening into an existing tuberculosis control program, where all patients initiating anti-tuberculosis treatment were routinely tested for diabetes using HbA1c within established diagnostics and reporting systems, demonstrated the potential to improve case detection among high-risk populations [43]. Similarly, community-based approaches, including home-based self-testing supported by a mobile application, reported in a preprint study, demonstrated favorable feasibility and acceptability in the study setting [45]. Key facilitators within service delivery included integration with existing programs, implementation of community outreach strategies (e.g., community-based screening camps, mobile diagnostic units, and household-level testing) and partial decentralization of services in some settings [43, 45]. However, these were often offset by fragmented care pathways, weak referral systems, and limited access to services at the primary care level. Delays between initial presentation and confirmatory diagnosis, combined with weak referral and follow-up systems, were associated with loss to follow-up across the care pathway, as reflected in care cascade analyses from a preprint national care cascade analysis showing low retention in care [42]. As a result, continuity from initial contact to diagnosis and follow-up was frequently disrupted.

### Health workforce

Health workforce capacity was reported as a critical determinant of early detection. Across studies, frontline healthcare providers represented the primary point of contact for patients, yet their ability to recognize early symptoms of T1DM was often limited. Evidence highlighted specific gaps in provider knowledge and training, particularly among newly trained physicians, including limited recognition of hypoglycemia symptoms and uncertainty in managing diabetes-related emergencies [39]. These gaps were partly attributed to insufficient clinical exposure during training and a lack of standardized curricula in diabetes education [39]. In many settings, pediatric diabetes care was concentrated among specialists, including pediatric endocrinologists, with limited diagnostic capacity at the primary care level [39, 40]. This contributed to delays in recognition and referral, especially in areas where specialist services were not readily accessible, and patients were initially managed by general practitioners with variable training [39, 40]. Some studies identified facilitators that could strengthen workforce capacity. Increased clinical exposure during training, case-based learning approaches, and structured education programs were associated with improved provider knowledge and reported competency [44, 46]. However, persistent shortages of trained personnel, lack of standardized training curricula, and limited opportunities for hands-on experience were major barriers. National assessments further highlighted gaps in the availability of trained staff at the facility level and inequitable distribution of workforce capacity across regions and facility types [41, 44]. These limitations likely reduced frontline providers' ability to recognize early symptoms and initiate timely referrals. In addition, limited caregiver and community awareness of early diabetes symptoms, including polyuria, polydipsia, and weight loss, may have further contributed to delays in presentation and diagnosis [40, 44].

### Health information systems

Health information systems influenced early detection primarily through their roles in patient tracking, data integration, and monitoring of care pathways. In some contexts, centralized laboratory systems, such as the South African National Health Laboratory Service (NHLS), integrated laboratory data across facilities and enabled longitudinal patient tracking through probabilistic record linkage, providing insights into diagnostic and care cascades reported in a preprint study [42]. Digital tools also supported specific implementation efforts across several studies. These included mobile applications guiding self-testing and result interpretation (e.g., HealthPulse TestNow™), tablet-based data collection platforms such as Research Electronic Data Capture (REDCap), and integrated digital reporting systems combining mobile applications with centralized laboratory management systems and management information systems for real-time monitoring and reporting reported in a preprint study [45], as well as a tuberculosis-diabetes screening program [43]. In addition, proposed models incorporated artificial intelligence–based screening and electronic health record (EHR)–supported data exchange within hub-and-spoke systems to improve diagnostic reach and efficiency [46]. However, most settings were characterized by fragmented and/or paper-based information systems with limited integration across levels of care. Inadequate data systems hindered patient tracking from initial presentation through diagnosis and follow-up, reducing opportunities for timely intervention and continuity of care.

### Access to essential medicines and technologies

Studies consistently highlighted gaps in the availability and affordability of basic diagnostic tools, including blood glucose testing and HbA1c measurement, particularly at the primary care level. Innovative approaches, such as point-of-care testing, mobile app–guided self-testing, and proposed AI-based screening models, demonstrated potential to expand access to diagnostics, especially in underserved areas [46]. These approaches emphasized the importance of bringing diagnostic capacity closer to the point of first contact. Despite these opportunities, implementation was often constrained by inconsistent supply chains, limited infrastructure, and affordability challenges. In many settings, limited access to reliable diagnostic tools necessitated reliance on clinical assessment, potentially contributing to delayed or inaccurate diagnoses and highlighting challenges in resource-constrained environments [47].

### Financing

Financing constraints were a major cross-cutting factor affecting early detection. High out-of-pocket costs for diagnostic tests were reported to limit access to care and contribute to delayed diagnosis [40]. Financial and logistical constraints, such as transport challenges and limited ability of caregivers to access services, were reported as barriers to accessing diagnostic services and follow-up care [40, 44]. Several facilitators were identified, including the inclusion of diabetes services within universal health coverage (UHC) packages and the presence of insurance mechanisms in certain settings. For example, in Kyrgyzstan, insulin was reported to be available in all public facilities under national coverage programs, and several countries had insurance mechanisms covering consultations and laboratory services [40]. However, these mechanisms were often insufficient to ensure equitable access, and coverage was inconsistent across populations and services. Dependence on external funding and donor-supported programs in several contexts raised concerns about the sustainability of early detection efforts and highlighted the need for more stable financing mechanisms.

### Leadership and governance

Leadership and governance structures shaped the broader environment for early detection. In several LMIC settings, including Kyrgyzstan, Mali, Peru, Tanzania, Kenya, and Malawi, national policies and strategies for non-communicable diseases (NCDs) were in place and served as a framework for improving diabetes care [40, 41, 44]. These policy environments acted as facilitators by enabling the inclusion of diabetes within national health priorities and UHC packages. However, implementation gaps were common. Early detection of diabetes was rarely operationalized as a distinct program area, and integration into primary care and community health systems remained limited. In several settings, reliance on vertical programs or external support led to fragmented implementation, with limited coordination across system levels. Opportunities for strengthening governance included better integration of diabetes into existing health programs, improved coordination among stakeholders, and greater emphasis on early detection in national strategies.

### Sensitivity analysis

A sensitivity analysis was conducted excluding the two preprint studies [42, 45] and the single-patient clinical narrative report [47]. The overall interpretation of the review remained largely unchanged. Health system barriers related to service delivery, health workforce capacity, access to diagnostics, financing, and governance continued to emerge consistently across the remaining studies. However, evidence relating to digital and home-based self-testing approaches, health information systems, and linkage-to-care became more limited following exclusion of these full-text articles. In particular, the evidence supporting care cascade analyses and patient tracking was substantially reduced because these findings were derived primarily from one excluded preprint study.

## Discussion

Early detection of T1DM in children remains challenging in many LMICs [22]. Across all health system components, early detection of pediatric T1DM appeared to be more strongly shaped by system capacity and implementation context rather than by discrete interventions. Some potentially promising approaches were identified, such as integration with existing programs, including one preprint study [42, 43] community-based testing reported in a preprint study, [45] and the use of digital tools [43, 45, 46]. These approaches were evaluated in a limited number of studies and were not implemented at scale. Facilitators such as program integration, [42, 43] policy support, [40, 44] and digital innovation [45, 46] suggest that improving early detection may be feasible within existing, resource-limited health systems. However, persistent barriers across the health system building blocks highlight the need for comprehensive, coordinated, system-level approaches that address multiple constraints simultaneously [40–42, 44]. The sensitivity analysis showed that these overall conclusions were unchanged, although evidence supporting digital and community-based implementation approaches became more limited after excluding the two preprint studies and the single-patient narrative report.

Across the included studies, several cross-cutting system challenges emerged. Early detection was largely passive and dependent on symptomatic presentation, which reflects the limited integration of screening into primary care. Poorly integrated referral and follow-up systems contributed to loss to follow-up across the diagnostic process. Workforce limitations, including gaps in training and expertise distribution issues, reduced the ability to recognize early symptoms at first contact. Lastly, insufficient access to diagnostics and financial barriers limited timely confirmation of diagnosis. These challenges highlight that delays in detection are driven by interconnected system weaknesses rather than isolated gaps [40–42, 44].

Simple tools such as blood glucose testing were not routinely available in primary care or community settings. This pattern reflects structural imbalances in service delivery, where diagnostic capacity remains concentrated at higher-level facilities, as reported in a preprint study [45]. In addition, delays between initial symptom detection and confirmatory diagnosis, combined with a weak referral and follow-up system, contributed to loss to follow-up [48]. Point-of-care testing and decentralized self-testing showed potential to expand access but were not widely implemented or scaled [45]. Although evidence specific to pediatric T1DM diagnosis in LMICs is limited, broader diabetes care literature suggests that the availability of diagnostic technologies alone may be insufficient to ensure high-quality care in primary care settings. Studies assessing diabetes care from other settings have reported that differences in quality of care may reflect lower adherence to clinical guidelines, limited provider experience, and inadequate staff training [49, 50].

Interventions were often implemented as vertical or disease-specific programs rather than integrated into broader health systems. For example, screening activities linked to tuberculosis programs demonstrated feasibility and, in some cases, high detection yield, but also highlighted the limitations of vertical models when not integrated into broader health systems. However, these approaches did not support sustained early detection due to fragmentation and poor coordination [43]. In contrast, horizontal approaches that integrate services within routine health systems may improve efficiency and sustainability [51]. Evidence suggests that early detection is more cost-effective when embedded within structured care pathways and integrated service delivery platforms. For instance, screening for T1DM can be combined with testing for celiac disease and other conditions detectable through blood-based assays [52]. These findings suggest that early detection of T1DM should be embedded within horizontal service delivery structures and, where feasible, combined with screening for other conditions to improve efficiency, feasibility, and sustainability.

Although this review focused on early detection, findings consistently highlighted gaps in linkage between detection, diagnosis, and care. Screening activities were rarely connected to structured referral or follow-up systems. As a result, children identified at risk were not consistently linked to confirmatory diagnosis or ongoing care. Care cascade analyses reported in a preprint study, showed substantial losses from diagnosis to retention and glycemic control [42]. Evidence from other disease programs underscores the importance of active linkage mechanisms, such as assisted referral, patient navigation, or follow-up contact, which are more effective than passive referral approaches in ensuring continuity of care [53, 54]. However, because evidence for linkage-to-care was substantially reduced in the sensitivity analysis, these findings should be interpreted cautiously until supported by additional peer-reviewed implementation studies. Financial and logistical barriers, including visit costs, transport costs, and caregiver constraints also limit successful linkage to care, reinforcing inequities in access to care in resource-constrained settings [55].

While we focused on health system factors, non-system determinants also play a crucial role in early detection. Caregiver education and health literacy play a critical role in recognizing early symptoms and seeking timely care. Limited awareness of symptoms such as polyuria, polydipsia, and weight loss contributes to delays in presentation and increased severity at diagnosis [56]. Although evidence is primarily derived from high-income settings, school-based interventions may offer an important platform for improving awareness among children, caregivers, and teachers and have been associated with earlier recognition and reduced severity at diagnosis in some settings [26]. However, whether these approaches are effective and feasible in LMICs remains uncertain.

### Limitations

This review has several limitations. First, the evidence base was limited and heterogeneous, with only nine studies meeting the inclusion criteria. To capture relevant health system evidence, the eligibility criteria were broadened during screening, potentially introducing selection bias. Two included studies were preprints, and one was a single-patient clinical narrative report. Although the sensitivity analysis indicated that the principal conclusions of the review remained largely unchanged after excluding these studies, evidence supporting some implementation approaches, health information system findings, and linkage-to-care became more limited. Second, few studies evaluated implemented interventions, preventing us from drawing conclusions regarding effectiveness and scalability. Third, some included studies were not exclusively focused on pediatric T1DM or did not distinguish between diabetes types, which may have affected the specificity of the findings. Lastly, the use of the WHO health system building blocks framework provided a structured approach to analysis but may not fully capture contextual factors influencing early detection, including social, behavioral, and community-level determinants.

## Conclusion

This systematic review found that evidence on early detection of pediatric T1DM in low- and middle-income countries remains limited, with only a small number of heterogeneous studies identified. Across the included studies, barriers were most consistently observed in service delivery, health workforce capacity, access to basic diagnostics, and referral systems, while evidence evaluating implemented strategies to improve early detection was scarce.

Improving early detection of pediatric T1DM in resource-constrained settings will require coordinated action across the diagnostic pathway rather than isolated interventions. Based on these findings, priority areas include expanding access to basic diagnostic tools at first-contact points, strengthening frontline provider capacity to recognize early symptoms, and embedding detection within routine primary care and existing service delivery platforms. Efforts should also strengthen referral and follow-up mechanisms and align financing to reduce access barriers. Future research should extend beyond descriptive studies toward the evaluation of scalable, system-integrated approaches that can be integrated within health systems and rigorously evaluated for effectiveness, feasibility, and sustainability in LMIC settings.

## Supplementary Information

Supplementary Material 1.

Supplementary Material 2.

Supplementary Material 3.

## Data Availability

All data generated or analyzed during this study are included in this article and its supplementary information files.

## Abbreviations

T1DM: Type 1 diabetes mellitus
LMICs: Low- and middle-income countries
DKA: Diabetic ketoacidosis
PRISMA: Preferred Reporting Items for Systematic Reviews and Meta-Analyses
PROSPERO: International Prospective Register of Systematic Reviews
IDF: International Diabetes Federation
MMAT: Mixed Methods Appraisal Tool
SANRA: Scale for the Assessment of Narrative Review Articles
WHO: World Health Organization
NCDs: Non-communicable diseases
TB–DM: Tuberculosis–diabetes mellitus
NHLS: National Health Laboratory Service
REDCap: Research Electronic Data Capture
EHR: Electronic health record
UHC: Universal health coverage
