# Supplementary material for: Health system barriers and pathways to strengthening early detection of pediatric type 1 diabetes in low- and middle-income countries: a systematic review
Source: BMC Public Health. 2026 Aug 3;26:3128. doi: 10.1186/s12889-026-28810-2 (PMC13721531; doi:10.1186/s12889-026-28810-2)
Supplement: Supplementary file 1 [file 12889_2026_28810_MOESM1_ESM.pdf]

Supplement 1: Search Strategy

PubMed:
#1
"Diabetes Mellitus, Type 1"[Mesh] OR "Diabetes Mellitus"[Mesh] OR "Diabetic Ketoacidosis"[Mesh] OR "type 1 diabet*"[tiab] OR "type I diabet*"[tiab] OR T1DM[tiab] OR "juvenile diabet*"[tiab] OR "pediatric diabet*"[tiab] OR "paediatric diabet*"[tiab] OR "childhood diabet*"[tiab] OR "diabet*"[tiab] OR "diabetic ketoacidosis"[tiab] OR DKA[tiab]
→ 979,936
#2
"Early Diagnosis"[MeSH Terms] OR "Mass Screening"[MeSH Terms] OR "early diagnos*"[Title/Abstract] OR "timely diagnos*"[Title/Abstract] OR "late diagnos*"[Title/Abstract] OR "delay* diagnos*"[Title/Abstract] OR "early detect*"[Title/Abstract] OR "symptom recogni*"[Title/Abstract] OR "case finding*"[Title/Abstract] OR "screen*"[Title/Abstract] OR "test*"[Title/Abstract] OR "new onset*"[Title/Abstract] OR "newly diagnose*"[Title/Abstract] OR "new onset*"[Title/Abstract] OR "new diagnose*"[Title/Abstract] OR "new diagnose*"[Title/Abstract] OR "new case*"[Title/Abstract] OR "detect*"[Title/Abstract] OR "diagn*"[Title/Abstract] OR "screen*"[Title/Abstract]
→ 10,171,692
#3
"Program Evaluation"[MeSH Terms] OR "Health Plan Implementation"[MeSH Terms] OR "Diffusion of Innovation"[MeSH Terms] OR "Implementation Science"[Title/Abstract] OR "intervention*"[Title/Abstract] OR "implement*"[Title/Abstract] OR "implementation"[Title/Abstract] OR "adopt*"[Title/Abstract] OR "uptake"[Title/Abstract] OR "rollout"[Title/Abstract] OR "scale-up"[Title/Abstract] OR "scale-up"[Title/Abstract] OR "adapt*"[Title/Abstract] OR "modifi*"[Title/Abstract] OR "tailor*"[Title/Abstract] OR "barrier*"[Title/Abstract] OR "facilitator*"[Title/Abstract] OR "challenge*"[Title/Abstract] OR "enabler*"[Title/Abstract] OR "obstacle*"[Title/Abstract] OR "effective*"[Title/Abstract] OR "impact*"[Title/Abstract] OR "outcome*"[Title/Abstract] OR "evaluat*"[Title/Abstract] OR "assess*"[Title/Abstract] OR "perform*"[Title/Abstract] OR "success*"[Title/Abstract] OR "sustainab*"[Title/Abstract]
→ 18,116,160
#4
"Developing Countries"[MeSH Terms] OR "developing countr*"[Title/Abstract] OR "lmic*"[Title/Abstract] OR "low income countr*"[Title/Abstract] OR "middle income countr*"[Title/Abstract] OR "low and middle income"[Title/Abstract] OR "low and middle income"[Title/Abstract] OR "lower-middle income"[Title/Abstract] OR "upper-middle

income"[Title/Abstract] OR "resource limit*"[Title/Abstract] OR "resource-constrained"[Title/Abstract] OR "resource-constrained"[Title/Abstract] OR "low-resource"[Title/Abstract] OR "low-resource"[Title/Abstract] OR "resource poor*"[Title/Abstract] OR "poor resource*"[Title/Abstract] OR "limited resource*"[Title/Abstract] OR "under-resourced"[Title/Abstract] OR "underserved"[Title/Abstract]
→ 283,414
#1 AND #2 AND #3 AND #4 → 3,888

Web of Science:
#1
AB("Diabetes Mellitus, Type 1" OR "Diabetic Ketoacidosis" OR "type 1 diabet*" OR "type I diabet*" OR "T1DM" OR "juvenile diabet*" OR "pediatric diabet*" OR "paediatric diabet*" OR "childhood diabet*" OR "Diabetic Ketoacidosis" OR "DKA")
→ 783,270
#2
AB("Early Diagnosis" OR "Mass Screening" OR "early diagnos*" OR "timely diagnos*" OR "late diagnos*" OR "delay* diagnos*" OR "early detect*" OR "symptom recogni*" OR "case finding*" OR screen* OR test* OR "new onset*" OR "newly diagnose*" OR "new diagnose*" OR "new case*" OR detect* OR diagn*)
→ 15,058,155
#3
AB("Program Evaluation" OR "Health Plan Implementation" OR "Diffusion of Innovation" OR "Implementation Science" OR intervention* OR implement* OR implementation OR adopt* OR uptake OR rollout OR "scale-up" OR adapt* OR modifi* OR tailor* OR barrier* OR facilitator* OR challenge* OR enabler* OR obstacle* OR effective* OR impact* OR outcome* OR evaluat* OR assess* OR perform* OR success* OR sustainab*)
→ 33,860,275
#4
AB("Developing Countries" OR "developing countr*" OR lmic* OR "low income countr*" OR "middle income countr*" OR "low and middle income" OR "lower-middle income" OR "upper-middle income" OR "resource limit*" OR "resource-constrained" OR "low-resource" OR "resource poor*" OR "poor resource*" OR "limited resource*" OR "under-resourced" OR underserved)
→ 353,355
#1 AND #2 AND #3 AND #4 → 3,335

Embase:
#1

'type 1 diabet*':ti,ab OR 'type i diabet*':ti,ab OR t1dm:ti,ab OR 'juvenile diabet*':ti,ab OR 'pediatric diabet*':ti,ab OR 'paediatric diabet*':ti,ab OR 'childhood diabet*':ti,ab OR diabet*:ti,ab OR 'diabetic ketoacidosis':ti,ab OR dka:ti,ab
→ 1,442,732
#2
'early diagnos*':ti,ab OR 'timely diagnos*':ti,ab OR 'late diagnos*':ti,ab OR 'delay* diagnos*':ti,ab OR 'early detect*':ti,ab OR 'symptom recogni*':ti,ab OR 'case finding*':ti,ab OR screen*:ti,ab OR test*:ti,ab OR 'new onset*':ti,ab OR 'newly diagnose*':ti,ab OR 'new diagnose*':ti,ab OR 'new case*':ti,ab OR detect*:ti,ab OR diagn*:ti,ab
→ 13,930,598
#3
intervention*:ti,ab OR implement*:ti,ab OR implementation:ti,ab OR adopt*:ti,ab OR uptake:ti,ab OR rollout:ti,ab OR 'scale-up':ti,ab OR adapt*:ti,ab OR modifi*:ti,ab OR tailor*:ti,ab OR barrier*:ti,ab OR facilitator*:ti,ab OR challenge*:ti,ab OR enabler*:ti,ab OR obstacle*:ti,ab OR effective*:ti,ab OR impact*:ti,ab OR outcome*:ti,ab OR evaluat*:ti,ab OR assess*:ti,ab OR perform*:ti,ab OR success*:ti,ab OR sustainab*:ti,ab
→ 24,126,565
#4
'developing countr*':ti,ab OR lmic*:ti,ab OR 'low income countr*':ti,ab OR 'middle income countr*':ti,ab OR 'low and middle income':ti,ab OR 'lower-middle income':ti,ab OR 'upper-middle income':ti,ab OR 'resource limit*':ti,ab OR 'resource-constrained':ti,ab OR 'low-resource':ti,ab OR 'resource poor*':ti,ab OR 'poor resource*':ti,ab OR 'limited resource*':ti,ab OR 'under-resourced':ti,ab OR underserved:ti,ab
→ 285,510
#1 AND #2 AND #3 AND #4 → 6,517

Cochrane (conducted July 2026):
#1
[mh "Diabetes Mellitus, Type 1"] OR [mh "Diabetes Mellitus"] OR [mh "Diabetic Ketoacidosis"] OR (type NEXT 1 NEXT diabet*):ti,ab OR (type NEXT I NEXT diabet*):ti,ab OR T1DM:ti,ab OR (juvenile NEXT diabet*):ti,ab OR (pediatric NEXT diabet*):ti,ab OR (paediatric NEXT diabet*):ti,ab OR (childhood NEXT diabet*):ti,ab OR diabet*:ti,ab OR "diabetic ketoacidosis":ti,ab OR DKA:ti,ab
→ 133,730
#2
[mh "Early Diagnosis"] OR [mh "Mass Screening"] OR (early NEXT diagnos*):ti,ab OR (timely NEXT diagnos*):ti,ab OR (late NEXT diagnos*):ti,ab OR (delay* NEXT diagnos*):ti,ab OR (early NEXT detect*):ti,ab OR (symptom NEXT recogni*):ti,ab OR (case NEXT finding*):ti,ab OR

screen*:ti,ab OR test*:ti,ab OR (new NEXT onset*):ti,ab OR (newly NEXT diagnose*):ti,ab OR (new NEXT onset*):ti,ab OR (new NEXT diagnose*):ti,ab OR (new NEXT diagnose*):ti,ab OR (new NEXT case*):ti,ab OR detect*:ti,ab OR diagn*:ti,ab OR screen*:ti,ab
→ 809,629
#3
[mh "Program Evaluation"] OR [mh "Health Plan Implementation"] OR [mh "Diffusion of Innovation"] OR "Implementation Science":ti,ab OR intervention*:ti,ab OR implement*:ti,ab OR "implementation":ti,ab OR adopt*:ti,ab OR uptake:ti,ab OR rollout:ti,ab OR "scale-up":ti,ab OR "scale-up":ti,ab OR adapt*:ti,ab OR modifi*:ti,ab OR tailor*:ti,ab OR barrier*:ti,ab OR facilitator*:ti,ab OR challenge*:ti,ab OR enabler*:ti,ab OR obstacle*:ti,ab OR effective*:ti,ab OR impact*:ti,ab OR outcome*:ti,ab OR evaluat*:ti,ab OR assess*:ti,ab OR perform*:ti,ab OR success*:ti,ab OR sustainab*:ti,ab
→ 1,858,262
#4
[mh "Developing Countries"] OR (developing NEXT countr*):ti,ab OR lmic*:ti,ab OR (low NEXT income NEXT countr*):ti,ab OR (middle NEXT income NEXT countr*):ti,ab OR "low and middle income":ti,ab OR "low and middle income":ti,ab OR "lower-middle income":ti,ab OR "upper-middle income":ti,ab OR (resource NEXT limit*):ti,ab OR "resource-constrained":ti,ab OR "resource-constrained":ti,ab OR "low-resource":ti,ab OR "low-resource":ti,ab OR (resource NEXT poor*):ti,ab OR (poor NEXT resource*):ti,ab OR (limited NEXT resource*):ti,ab OR "under-resourced":ti,ab OR underserved:ti,ab
→167,38
#1 AND #2 AND #3 AND #4 → 633

Targeted grey literature search (conducted July 2026):
Sources searched:

- World Health Organization IRIS repository (https://iris.who.int/)
- International Diabetes Federation website (https://idf.org/)

Search terms included:

1. “type 1 diabetes” children diagnosis
2. “type 1 diabetes” diabetic ketoacidosis children
3. “type 1 diabetes” primary care
4. “type 1 diabetes” low resource
5. "type 1 diabetes" implementation
6. "type 1 diabetes" screening
