# Supplementary material for: Health system barriers and pathways to strengthening early detection of pediatric type 1 diabetes in low- and middle-income countries: a systematic review
Source: BMC Public Health. 2026 Aug 3;26:3128. doi: 10.1186/s12889-026-28810-2 (PMC13721531; doi:10.1186/s12889-026-28810-2)
Supplement: Supplementary file 2 [file 12889_2026_28810_MOESM2_ESM.docx]

**Supplement 2**

Supplementary Table S1. Mapping of synthesized themes to the WHO health system building blocks and stages of the pediatric T1DM diagnostic pathway

| **WHO health system building block** | **Community awareness & symptom recognition** | **First healthcare contact** | **Diagnostic evaluation & confirmation** | **Referral** | **Linkage to care** | Supporting studies* |
| --- | --- | --- | --- | --- | --- | --- |
| **Service Delivery** | Community outreach | Passive, facility-based detection | Centralized diagnosis and delayed confirmation | Fragmented referral | Weak follow-up | 42, 43, 45 |
| **Health Workforce** | Limited caregiver awareness | Insufficient frontline training | Limited specialist expertise | Delayed referral | Workforce capacity constrains | 39, 40, 41, 44 |
| **Health Information Systems** | -- | Fragmented information systems | Digital reporting and laboratory systems | Limited patient tracking | Poor continuity of care | 42,43,45 |
| **Access to Essential Medicines & Technologies** | -- | Limited diagnostic availability | Point-of-care, self-testing, and AI-supported diagnostics | -- | -- | 40, 45, 46, 47 |
| **Financing** | -- | Financial access barriers | Out-of-pocket diagnostic costs | Financial barriers | Financial and logistical barriers | 40, 44 |
| **Leadership & Governance** | National NCD policies | Limited integration into primary care | No dedicated early detection strategy | Fragmented coordination | Weak policy implementation | 40,41,44 |

* Supporting studies contributed evidence to one or more synthesized themes within each WHO health system building block and are not intended to correspond to every individual cell.
