# Supplementary material for: Health system barriers and pathways to strengthening early detection of pediatric type 1 diabetes in low- and middle-income countries: a systematic review
Source: BMC Public Health. 2026 Aug 3;26:3128. doi: 10.1186/s12889-026-28810-2 (PMC13721531; doi:10.1186/s12889-026-28810-2)
Supplement: Supplementary file 3 [file 12889_2026_28810_MOESM3_ESM.docx]

**Supplement 3**

Table S2. Methodological Quality Assessment of Included Studies Using the Mixed Methods Appraisal Tool (MMAT)

| Mixed Methods | | | | | | | |
| --- | --- | --- | --- | --- | --- | --- | --- |
| Author (Year) | S1. Clear research questions? | S2. Data allow addressing question? | Is there an adequate rationale for using a mixed methods design to address the research question? | Are the different components of the study effectively integrated to answer the research question? | Are the outputs of the integration of qualitative and quantitative components adequately interpreted? | Are divergences and inconsistencies between quantitative and qualitative results adequately addressed? | Do the different components of the study adhere to the quality criteria of each tradition of the methods involved? |
| Aida Abdraimova (2022) | Yes | Yes | Yes | Yes | Yes | No | No |
| Abeer Alassaf (2023) | Yes | Yes | Yes | Yes | Yes | No | No |
| Quantitative non-randomized | | | | | | | |
| Author (Year) | S1. Clear research questions? | S2. Data allow addressing question? | Are the participants representative of the target population? | Are measurements appropriate regarding both the outcome and intervention (or exposure)? | Are there complete outcome data? | Are the confounders accounted for in the design and analysis? | During the study period, is the intervention administered (or exposure occurred) as intended? |
| Abeer Alassaf (2023) | Yes | Yes | No | Yes | Yes | Yes | Yes |
| Rita Ammoun (2022) | Yes | Yes | Yes | Yes | Yes | Yes | Yes |
| Alana T Brennan (2025) | Yes | Yes | No | Yes | Yes | Yes | Yes |
| Shifa Salman Habib (2020) | Yes | Yes | No | Yes | Yes | Yes | Yes |
| Carolyn Oliver (2025) | Yes | Yes | No | Yes | Yes | Yes | Yes |
| Mrinmoy Roy (2025) | Yes | Yes | No | Yes | Yes | Yes | Yes |

Table S3: Quality Appraisal of Narrative Review Using the SANRA Tool

| First Author (Year) | Title | Importance justified (0-2) | Clear aims/questions (0-2) | Literature search described (0-2) | Referencing (0-2) | Scientific reasoning (0-2) | Appropriate data presentation (0-2) | Total SANRA score (0-12) | Overall appraisal |
| --- | --- | --- | --- | --- | --- | --- | --- | --- | --- |
| van Heerwaarde (2021) | Approach to Diagnosing a Pediatric Patient with Severe Insulin Resistance in Low- or Middle-income Countries | 2 | 2 | 0 | 2 | 2 | 2 | 10 | High quality narrative review; main limitation is absence of described literature search |
